# The value of qualitative methods to public health research, policy and practice

**DOI:** 10.1177/17579139221083814

**Published:** 2022-04-01

**Authors:** T Stickley, A O’Caithain, C Homer

**Affiliations:** Institute of Mental Health, University of Nottingham Faculty of Medicine and Health Sciences, Triumph Road, Nottingham NG7 2UH, UK; The University of Sheffield, Sheffield, UK; Sheffield Hallam University, Sheffield, UK

**Keywords:** public health, qualitative methods, publishing

## Abstract

This article reviews the role and use of qualitative methods in public health research.

‘Signs of quality’ are introduced to help guide potential authors to publish their qualitative research in public health journals. We conclude that high-quality qualitative research offers insights that quantitative research cannot. It is time for all public health journals to recognise the value of qualitative research and increase the amount that they publish.

## Introduction

In this article, we briefly review the role and use of qualitative methods in public health research and its significance for research, policy and practice. Historically, public health research has been largely dependent on quantitative research rooted in medical science. Qualitative research approaches, however, are able to provide the ‘lived experience’ perspective of patients, practitioners and the public on any aspect of public health.

To inform this article, we searched the most recent original research articles published in ten of the most widely cited public health journals in the world (generally those with the highest impact factor, including *Perspectives in Public Health*). The list of journals can be found in Table 1.

**Table 1:** The methods used in 100 recently published original research articles in 10 public health journals

Journal	Quantitative	Mixed-methods	Qualitative
*American Journal of Public Health*	10	0	0
*Annual Review of Public Health*	10	0	0
*BMC Public Health*	9	0	1
*European Journal of Public Health*	10	0	0
*Frontiers in Public Health*	6	3	1
*International Journal of Public Health*	8	1	1
*Journal of Public Health*	7	3	0
*Perspectives in Public Health*	8	1	1
*Public Health*	7	3	0
*The Lancet Public Health*	10	0	0
TOTAL	85	11	4

We examined 100 of the most recently published original research articles (10 from each journal up until May, 2021) to discover how many of these reported qualitative methods. The findings from this quick review can be found in Table 1 below. The review revealed that 85 articles reported quantitative methods, 11 reported mixed-methods, and only 4 reported qualitative methods. In our review, we deliberately did not include one public health journal, *Critical Public Health* because it specialises in publishing qualitative public health research studies. With only four qualitative research papers out of the most recent 100 public health original research articles published in the top journals, we have decided to publish this article first to encourage qualitative research practices in public health, second to highlight the value of qualitative research, third to briefly identify what makes ‘good qualitative research’ and finally to promote increased submissions of original qualitative research in this and other public health journals.

## Reporting Qualitative Health Research

Qualitative research has its origins in Interpretivism. As such, it has been widely used in the social sciences, in contrast to the medical sciences that historically have largely embraced the positivist tradition. Typically, public health research has followed the positivist tradition although qualitative research methodology appears more often in public health journals than top medical journals. For example, a cursory examination of the Lancet indicates that it does not appear to publish any qualitative research and the *British Medical Journal* (*BMJ*) rarely does so. In 2016, the *BMJ* published an open letter from 76 senior academics from 11 countries inviting its editors to: ‘*… reconsider their policy of rejecting qualitative research on the grounds of low priority. They challenge the journal to develop a proactive, scholarly, and pluralist approach to research that aligns with its stated mission*’.^1^ Included in their support for qualitative research articles in the *BMJ*, they observe that many of the journal’s top papers have been qualitative studies. This letter has been cited 250 times in the literature, largely supportive of their views. In their reply to the letter, Editors of the *BMJ* acknowledge that: ‘*… we agree they can be valuable, and recognise that some research questions can only be answered by using qualitative methods*’.^2^ In so much as we can tell to date, the *BMJ* has not changed its practice. Fortunately, published accounts of qualitative research in various other health disciplines flourishes, for example, there are now at least two health journals that are exclusively designed for this purpose (*Qualitative Health Research* and *International Journal of Qualitative Studies on Health and Well-being*).

## The Value of Qualitative Health Research

The following quotation succinctly argues the need for qualitative research methods in public health:

*Public health, we believe, needs both epidemiology and qualitative research. Without epidemiology we cannot answer questions about the prevalence of and association between health determinants and outcomes. Without qualitative enquiry, it is difficult to explain how individuals interpret health and illness in their everyday lives, or to understand the complex workings of the social, cultural and institutional systems that are central to our health and wellbeing.* ^3^

In particular, given a situation with complex phenomena involving human experience and behaviour, quantitative research may equally excel in finding out ‘what and when?’, but qualitative research may equally be needed to find out ‘why, how and how come?’. Green and Britten^4^ summarise the role of qualitative research in health, and we have adapted their key points to apply to public health:

- Qualitative methods can help bridge the gap between scientific evidence and public health policy and practice by investigating human perceptions and experiences.
- Recognising the limits of quantitative approaches and that different research questions require different kinds of research.
- Qualitative research findings provide rigorous and firsthand accounts of public health educational, promotional and clinical practices in everyday contexts.
- Qualitative research can be used to help inform individual health choices and health promotion initiatives within communities.

## Doing High-Quality Qualitative Research

Quality is unlikely to be the only reason that so little qualitative research finds its way into public health journals; even research articles of the highest quality may be met with resistance from reviewers and editors. Nonetheless it is important to attend to quality. Articles using qualitative methods require the same rigour as articles reporting quantitative methods; however, the criteria for assessing rigour are different. When assessing qualitative articles, we need to remember that what is considered rigorous in the social sciences is not necessarily the same as what is considered rigorous in the medical sciences and vice versa. Either way, what is important is that public health journals publish high-quality research studies, whatever methodology is employed. The following quotation is helpful in focusing on the need for rigour in qualitative approaches to healthcare research:

*The use of qualitative research in health care enables researchers to answer questions that may not be easily answered by quantitative methods. Moreover, it seeks to understand the phenomenon under study in the context of the culture or the setting in which it has been studied …* (however this) *… requires researchers in health care who attempt to use it, to have a thorough understanding of its theoretical basis, methodology and evaluation techniques.*^5^

As quoted above, Al-Busaidi,^5^ asserts that qualitative health researchers need an appreciation of theory and methodologies and use of both in all research and evaluation studies. What is most important in any qualitative study is that the research question is clear and the method is appropriate to answer the research question. We can therefore begin to ask critical questions of any qualitative article submitted for publication in public health journals:

- Is the research question clear?
- Is the method appropriate for addressing the research question?
- Is there an explanation as to how and why this method is appropriate?
- What are the theories referred to in this study and how are these applied?
- Are these theories consistent throughout the study?
- Has the sample been critiqued to make readers aware of who is not included and how this might affect findings?
- Is the analysis grounded in the data?
- Does the analysis address questions of the data so that insights are identified that go beyond simply describing what participants have said?
- Are there clearly articulated implications for public health practice?

In addition to these fundamental questions, to help researchers report qualitative research, there are two frameworks that help to maintain standards for the conduct and reporting of the method. The first is COREQ (Consolidated criteria for reporting qualitative research).^6^ This is a 32-point checklist of three domains: research team and reflexivity, study design and analysis and findings. The second is Standards for Reporting Qualitative Research (SRQR),^7^ which is a 21-point check-list following the same format. Together, these are both useful tools for helping researchers think about what they need to consider when conducting qualitative research and for helping reviewers assess articles using qualitative methods. We are not suggesting that qualitative researchers should use these frameworks as tick-box checklists, although they may be used to enable researchers to think through important elements of qualitative research that may be otherwise overlooked. At the end of this article, we supply weblinks to enable the reader to inspect these two frameworks.

## ‘Signs of Quality’ for Reporting Qualitative Public Health Research

Rather than leave the reader baffled by frameworks and checklists, we propose a number of ‘signs of quality’ that we would expect to see when reviewing articles submitted to this or any other high-quality public health journal.

### Focussed

The research question is clearly identified and clearly related to public health policy or practice and the chosen method is appropriate for answering that question. A rationale is offered to justify the study and the methods used.

### Ethical

Ethical questions are considered, the study has been conducted and reported in an ethical manner, and ethical approval has been granted from a recognised ethics committee.

### Clear

How the study was implemented needs to be reported as clearly as possible including: how access to participants was achieved, what questions were asked, and how the analysis was conducted.

### Consistent

The study needs to be both theoretically and practically consistent. For example, if the study claims to be narrative research, did the questions elicit stories and is narrative theory used in analysis?

### Collaborative

In recent years, health services in many countries have embraced patient and public involvement and co-production in both research and practice. Such initiatives are designed to draw our attention to service users’ views, needs and desires. This agenda sits very well with qualitative research methodologies.

### Contribution

Every research study needs to make a contribution to the body of knowledge concerning the subject under investigation. If there is theoretical and practical consistency throughout the study and it has been competently conducted and analysed, the reader should come away with a sense of learning something new on the topic. This insight should be easy for a reader to take away from each article and the easiest way to do this is to articulate it clearly in the conclusion in the abstract as well as the conclusion in the body of the paper. Conclusions of ‘it’s complex’ or ‘there were five issues affecting this phenomenon’ fail to offer useful insights. They may be a signal of an under-analysed study. It will be much more helpful to readers to state a single key issue that adds to the evidence base and that helps members of the population, policy-makers, or practitioners to understand the phenomenon under study or take action on it.

## Examples of Good Qualitative Research from this Journal

In order to exemplify the principles, we espouse in this article, we refer to two recent articles published in *Perspectives in Public Health* that use qualitative methods. First, Lozano-Sufrategui et al.^8^ aimed to ‘… *understand the behaviour changes men who attended a weight loss programme engage in during weight maintenance …*’. To achieve this aim, the research team encouraged men on a weight loss programme to keep photo-diaries of themselves and to talk about their progress with the researchers. The research is innovative in its approach and uniquely reports the participants’ thoughts, feelings and behaviours. It highlights the importance of drawing on the diversity of methods that exist beyond face-to-face interviews. The second example is Eley et al.^9^ who conducted interviews and focus groups in four countries in order to ‘*… explore school educators’ attitudes, behaviours and knowledge towards food hygiene, safety and education*.’ Using this approach, they were able to explore individual and group views on this subject thus identifying not only the need for more educational resources but barriers and opportunities in the process. While reading these articles, it becomes immediately apparent that these studies were able to gain insight into the respective topics that quantitative methods could never achieve. What qualitative research facilitates is the human connection between interviewer and interviewee and in that process, together with the guarantee of confidentiality, people are able to speak in-depth about their experiences and perceptions, from which much can be learned. In these two examples, the qualitative findings give insights into the thoughts and feelings of the participants and enable a greater understanding of how the researchers were able to draw their conclusions from the research.

## Conclusion

A review of top public health journals identified that the vast majority of research that is being currently published in high-ranking public health journals use quantitative methods. High-quality qualitative research offers insights that quantitative research cannot. It is time for all public health journals to recognise the value of qualitative research and increase the amount of high-quality qualitative research that they publish.

*COREQ link*:

*http://cdn.elsevier.com/promis_misc/ISSM_COREQ_Checklist.pdf*

*SRQR link*:

*https://onlinelibrary.wiley.com/pb-assets/assets/15532712/SRQR_Checklist-1529502683197.pdf*
